# A novel murine model of Sjögren’s disease using lacrimal autoantigen

**DOI:** 10.3389/fimmu.2026.1586519

**Published:** 2026-02-13

**Authors:** Siyuan Li, Shan Liu, Yaqiong Li, Peng Zhang, Fengyang Lei, Lei Tian, Ying Jie

**Affiliations:** 1 Beijing Institute of Ophthalmology, Beijing Tongren Eye Center, Beijing Key Laboratory of Ophthalmology and Visual Sciences, Beijing Tongren Hospital, Capital Medical University, Beijing, China; 2 Department of Ophthalmology, Beijing Fuxing Hospital, Capital Medical University, Beijing, China; 3 Beijing Advanced Innovation Center for Big Data-Based Precision Medicine, Beihang University and Capital Medical University, Beijing, China

**Keywords:** autoimmune, dry eye, inflammation, lacrimal gland, Sjögren’s disease

## Abstract

**Purpose:**

Sjögren’s disease (SjD) is a group of chronic autoimmune diseases primarily targeting exocrine glands, including the lacrimal glands (LG). Involvement of the lacrimal glands leads to severe dry eye, also known as Sjögren’s disease-associated dry eye (SjD-DE). Current, available animal models of SjD are achieved by using autoantigens from salivary gland. This study establishes a novel lacrimal gland-specific autoimmune model that recapitulates key features of SjD-DE, providing a tool for investigating LG-focused mechanisms in SjD.

**Methods:**

To establish the lacrimal gland-specific Sjögren’s model (termed LGSS) model, autoimmune responses were induced in mice using homogenized lacrimal gland proteins. LGSS mice were evaluated at various timepoints after immunization to determine SjD development. SjD phenotype such as tear and saliva secretion, lymphocyte infiltration in the lacrimal and salivary glands and serum autoantibody levels was assessed. Immune cell profiles in the spleen and cervical lymph nodes were evaluated via flow cytometry. In addition, corneal epithelial intactness, goblet cell density, lacrimal gland injury was evaluated to assess lacrimal gland involvement and secondary ocular surface damage. RNA sequencing and gene enrichment analysis of diseased lacrimal glands were performed.

**Results:**

LGSS mice demonstrated a reduced tear and saliva secretion, increased lymphocyte infiltration, and elevated autoantibody levels that are similar to common SjD mice. Additionally, the established LGSS mice demonstrated increased populations of Th1 and Th17 cell, along with lacrimal gland and ocular surface damage. RNA sequencing revealed that LGSS mice shared a common genetic profile with NOD mice, the spontaneous SjD model, such as *Parp9*, *Cdkn2c*, and *Ifi35*. Additionally, LGSS mice exhibited several uniquely expressed genes, including metabolism-related genes (*Cbs*, *Dlst*, *Sardh*) and genes associated with cellular processes (*Actc1*, *Tnnc1*).

**Conclusion:**

The LGSS mice have been shown that successfully replicates several key features of SjD and demonstrates significant lacrimal gland and ocular surface damages, making it a valuable animal model to study SjD-DE.

## Introduction

Sjögren’s disease (SjD) is a systemic autoimmune disorder characterized as progressive damage to exocrine glands. Involvement of the lacrimal glands leads to dry eye, which is often the early-onset symptom in patients with SjD (1). The incidence of SjD is approximately 0.6% (ranging from 0.19% to 1.39%), with the incidence in females being 14 times higher than that in males (2, 3). Studies reported that about 85% of all SjD patients reported with or experienced dry eye symptoms among the 1,208 participants registered in the International Sjögren’s Syndrome Registry, which is defined as Sjögren’s disease -associated dry eye(SjD-DE) (4). SjD-DE constitutes a major component of aqueous-deficient dry eye (ADDE) (5). In patients with severe ADDE, approximately 10% are concurrently diagnosed with SjD (5). Common symptoms of SjD-DE, other than dry eyes, also have irritation, pain, photophobia, foreign body sensation, and so on. Patients with SjD-DE experience more severe ocular symptoms, faster disease progression, and a higher risk of blindness in advanced stages compared to those with non- Sjögren’s disease -associated dry eye (NSDE).

To better understand the pathophysiology of SjD and develop potential management approaches, various animal model have been established, broadly categorized into spontaneous (e.g., NOD, NOD.B10, MRL/lpr mice) and induced models (e.g., Ro60/M3R peptide immunization, salivary gland homogenate-induced) (6). Spontaneous models, such as NOD mice, recapitulate systemic autoimmune features of SjD, including both lacrimal and salivary gland dysfunction, albeit with delayed onset (typically >20 weeks) (7). In contrast, induced models often prioritize salivary gland pathology (e.g., using submandibular gland antigens), while lacrimal gland involvement remains less characterized (8). Although some models (e.g., C57BL/6.NOD-Aec1Aec2) exhibit SS-like dry eye, their temporal dissociation (e.g., tear hyposecretion preceding gland inflammation) complicates mechanistic studies of early SjD-DE (9).

This study establishes a novel experimental lacrimal gland-specific Sjögren’s model (termed LGSS) using lacrimal homogenate as the autoantigen. Unlike systemic antigen-induced models (e.g., Ro60/M3R), LGSS directly targets lacrimal tissue, enabling focused investigation of SjD-DE pathogenesis. We hypothesize that LGSS mice will mirror key SjD-DE features-rapid tear reduction, lymphocytic infiltration, and ocular surface damage-while complementing existing models for studying organ-specific autoimmunity.

## Methods

### Animals

Female NOD/ShilLtJGpt mice (Strain NO. N000235) aged 8 and 12 weeks were purchased from GemPharmatech (Nanjing, China) (Hereinafter referred to as NOD8wk and NOD12wk.). Female C57BL/6N mice aged 6–8 weeks were obtained from Charles River Laboratory Animal Center (Beijing, China). They were housed under standard conditions at the Animal Center of Capital Medical University, provided with a standard diet, and maintained on a 12-hour light-dark cycle. All animal experiments were conducted in accordance with the guidelines of the Association for Research in Vision and Ophthalmology (ARVO) for the use of animals in ophthalmic and vision research (10). The relevant procedures were approved by the Ethics Committee for Animal Research of Capital Medical University (Ethics Number: AEEI- 2022-233).

### Animal model establishment

Six-week-old female C57BL/6N mice were used to establish the model (Hereinafter referred to as LGSS). The lacrimal glands were excised, homogenized, and the supernatant was collected to measure protein concentration. On days 0 and 7, the lacrimal gland proteins were mixed with Freund’s complete adjuvant (#F5881, Sigma-Aldrich) in a 1:1 ratio, resulting in a final concentration of 4 mg/mL. The LGSS mice were depilated on their necks, and 100 μL of the antigen mixture was injected subcutaneously at 5–6 different points. On day 14, Freund’s incomplete adjuvant (#F5506, Sigma-Aldrich) was mixed in a 1:1 ratio to achieve a final concentration of 2 mg/mL, and the same volume was subcutaneously injected into the necks of the LGSS mice. The control group received PBS buffer injections following the same protocol in terms of timing, site, and volume. LGSS and control mice were analyzed at 21 days post-immunization (acute phase), while NOD mice were assessed at 8 and 12 weeks of age to span pre-symptomatic and early symptomatic stages.

### Tear secretion measurement

Tear volume was measured by phenol red cotton thread(PRT), as described (11). Briefly, after deep anesthesia of the mice, a PRT was inserted into the lateral canthus. After 1 minute, the thread was removed, and the wet length was recorded as the tear secretion volume. This process was repeated three times, and the average was calculated.

### Saliva secretion measurement

After deep anesthesia of the mice, 0.04 mg/mL pilocarpine hydrochloride (#HY-B0726, Med Chem Express) was injected intraperitoneally at a dose of 0.5 mg/kg. After 5 minutes, pre-weighed filter paper was inserted into the opening of the submandibular gland, and the mice were positioned with their heads lower than their feet. After 15 minutes, the filter paper was removed and weighed, with the difference in weight recorded as the saliva secretion volume. The relative salivary flow rate was calculated as follows: relative salivary flow rate (mg/min/g) = saliva mass (mg)/[collection time (min) × body weight (g)].

### Histology and immunofluorescence analysis

After euthanizing the mice in each group, the eyeballs, lacrimal glands, and salivary glands were harvested. The tissues were rinsed with PBS buffer and stored in 4% paraformaldehyde. Both paraffin and frozen sections were prepared accordingly. Standard protocols were followed for hematoxylin and eosin (HE) staining of paraffin-embedded sections of lacrimal and salivary glands, which were then scanned for imaging. A lymphocytic infiltration focus containing more than 50 lymphocytes was defined, and the number of lymphocytic infiltration foci was counted. Subsequently, sagittal sections of the eyeball were subjected to periodic acid-Schiff (PAS) staining, and the number of positive cells in the conjunctival sac was counted.

For the detection of apoptosis, frozen sections of lacrimal gland tissues were stained using a one- step TUNEL assay kit (#E-CK-A320, Elabscience) following the manufacturer’s instructions. Imaging was performed using a Leica DMi8 confocal laser microscope. ImageJ software was used to count the total number of cells and the number of positive cells in each image, calculate the apoptosis rate, and perform statistical analysis.

The procedure for immunofluorescence on frozen sections was carried out as described (12). Briefly, the frozen sections were first fixed with 4% paraformaldehyde for 30 minutes, followed by permeabilization with 0.3% Triton for 10 minutes. The sections were then blocked with 10% goat serum for 1 hour and incubated with primary antibodies overnight at 4°C. The primary antibodies used included: AQP5(1:100, #20334, Proteintech), and ACTA2(1:200, #67735, Proteintech). The next day, secondary antibodies (Invitrogen) were incubated at 37 °C for 1 hour in the dark. Finally, the sections were mounted using DAPI Fluoromount-G (#0100, Southern Biotech). Imaging was performed using a Leica DMi8 confocal laser microscope. The fluorescence intensity was measured using ImageJ software.

### Serum autoantibody level detection

After deeply anesthetizing the mice, their whiskers were trimmed, and blood was collected by enucleating the eyes. The whole blood was allowed to stand at room temperature for 2 hours, followed by centrifugation at 10,000 rpm for 15 minutes at 4 °C. The supernatant serum was then collected, aliquoted, and stored at -80 °C until analysis. Detection was performed using Mouse Anti-SSA/SSB Total Ig’s ELISA Kit (#5710/5810, Alpha Diagnostic International) according to the manufacturer’s instructions. Finally, the Positive Index was used for statistical analysis.

### Corneal fluorescein staining and slit-lamp photography

After deep anesthesia of the mice, 1 μL of a 1 mg/mL fluorescein sodium solution was instilled into the conjunctival sac of the mice. Once applied to the ocular surface, excess liquid was carefully wiped away. Using a yellow filter, cobalt blue light from the slit lamp (BX900, HAAG-STREIT) was applied to observe corneal epithelial staining, and photographs were taken. The stained area of fluorescein sodium was quantified as a proportion of the entire corneal surface using ImageJ software.

### Flow cytometric analysis

Spleen and cervical lymph nodes were harvested and mechanically dissociated into single-cell suspensions. Surface staining was performed before intracellular staining. Antibodies to the following were used: to mouse CD3 APC/Fire 750(BioLegend, Clone 17A2), CD4 BV421(BioLegend, Clone GK1.5), IFN-γ PerCP/Cy5.5(BioLegend, Clone XMG1.2), IL-4 PE(BioLegend, Clone 11B11), IL-17A PE/Cy7(BioLegend, Clone TC11-18H10.1), CD25 PE(BioLegend, Clone 3C7), Foxp3 APC(eBioscience, Clone FJK-16s). For intracellular staining, cells were fixed and permeabilized using a Foxp3/Transcription Factor Staining Buffer Set (#00-5523, eBioscience). The stained cells were analyzed using a BD LSRII Flow Cytometer (BD Biosciences), and data were processed with FlowJo software (Tree Star). Unstained cells were used to adjust the instrument settings to account for cellular autofluorescence or background fluorescence. The frequencies of Th1, Th2, Th17, and Treg cells were calculated as the percentage of CD4^+^ T cells expressing the respective markers.

### RNA isolation and RT-qPCR

For lacrimal glands, total RNA was isolated with Trizol reagent (#10606, YEASEN). RT-qPCR was conducted as described (12). First, cDNA Synthesis SuperMix (#11141, YEASEN) was used to reverse transcribe 1 µg RNA into cDNA. RT-qPCR was then conducted with the qPCR SYBR Green Master Mix (#11184, YEASEN) on a 7500 Real-time PCR system (Applied Biosystems, USA). **Supplementary Table S1** shows information about the primer sequences.

### RNA sequencing

Total RNA was extracted from lacrimal glands, and sequencing libraries were constructed according to previously described protocols (12). The RNA-Seq reads were mapped to the mouse reference genome GRCm38 using HISAT2 with default settings. Differential expression analysis was performed using DESeq2 to identify genes that were differentially expressed (DEGs) between the groups, with a |log2 fold change| ≥ 1 and a P-value < 0.05 as the criteria for significance. Principal component analysis (PCA) was first performed on the expression data to evaluate global transcriptomic variations between groups, and a heatmap was generated to illustrate expression patterns among the groups. To further explore the biological changes in LGSS mice, differential expression analysis was performed by comparing each of the three model groups with the control group, followed by GO and KEGG pathway analysis to uncover potential molecular mechanisms.

### Statistical analysis

Data analysis was conducted using GraphPad Prism 10 software. All datasets were first assessed for normality using the Shapiro-Wilk test. For normally distributed data involving multiple groups, we applied one-way ANOVA (for single-factor comparisons) or two-way ANOVA (for Group x Time interactions), followed by Tukey’s *post hoc* tests when significant main effects were detected. Non-normally distributed data were analyzed using the Kruskal-Wallis test with Dunn’s *post hoc* correction for multiple comparisons. Two-group comparisons utilized Student’s t-tests (normal data) or Mann-Whitney U tests (non-normal data). Results are presented as mean ± SD for parametric tests or median with interquartile range for nonparametric tests. Statistical significance was set at P < 0.05.

## Results

### LGSS mice exhibit SjD phenotype

**Figure 1:**
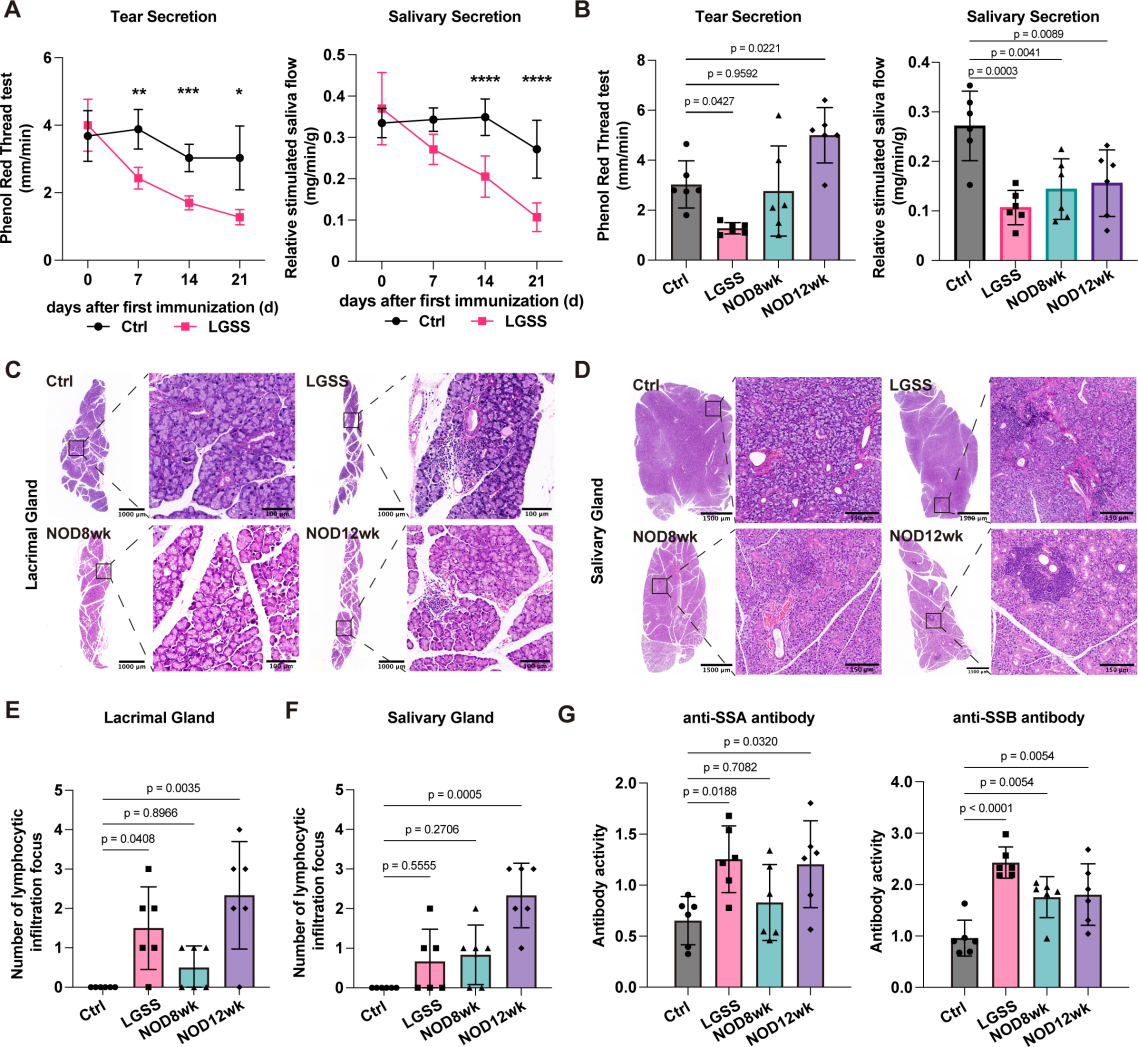
Establishment and characterization of the LGSS animal model. **(A)** Trends in tear secretion and saliva secretion during LGSS modeling process. **(B)** Tear secretion and saliva secretion at the end of the experiment in each group. **(C, D)** HE-stained sections of the lacrimal and salivary glands, with enlarged views of glandular structures (Scale bars, 1000 μm, 100 μm, 1500 μm and 150 μm, respectively). **(E, F)** Number of lymphocytic infiltration foci in lacrimal gland sections and submandibular gland sections. **(G)** Serum levels of anti-SSA and anti-SSB antibodies. (*p<0.05; **p<0.01; ***p<0.001; ****p<0.0001, n=6 mice/group).

Tear and saliva secretion levels were measured before model induction, and on days 7, 14, and 21 after the first immunization. The LGSS mice exhibited a gradual decline in both tear and saliva secretion (**Figure 1A**). Tear secretion showed a significant difference compared to the control group on days 7, 14, and 21. In terms of saliva secretion, a significant difference was observed between the LGSS and control groups on days 14 and 21. After the LGSS model was established, we compared the tear and saliva secretion levels across the four groups. Comparison between NOD mice and the control group revealed no significant difference in tear secretion in 8-week-old NOD mice, whereas an increase in tear secretion was observed in 12-week-old NOD mice. In terms of saliva secretion, both displayed significantly lower levels compared to the control group (**Figure 1B**).

In the control group, both lacrimal glands and submandibular glands display well-organized structures, with distinct serous acini and ducts arranged in lobules. The acinar cells are pyramid-shaped, containing round nuclei, and the ducts are lined with cuboidal epithelial cells. However, in the LGSS mice and 12-week-old NOD mice, the glands exhibit a disorganized tissue structure, with enlarged acini and irregular lobule spacing. However, morphological changes were rarely observed in the glands of 8-week-old NOD mice (**Figures 1C, D**). Lymphocytic infiltration foci were significantly increased in the lacrimal glands of LGSS and 12-week-old NOD mice compared to the control group (**Figure 1E**), but only 12-week-old NOD mice had significantly more foci in the salivary glands (**Figure 1F**).

Positive index was used to describe serum autoantibodies levels. A sample value would be positive if significantly above the value of the determined non-immune panel, tested at the same sample dilution. For anti-SSA/Ro antibodies, the serum levels in LGSS and 12-week-old NOD mice were significantly higher than those in the control group, while no statistical difference was observed in 8-week-old NOD mice. For anti-SSB/La antibodies, serum levels were significantly elevated in all three groups (**Figure 1G**).

### LGSS mice have ocular manifestations similar to DED

**Figure 2:**
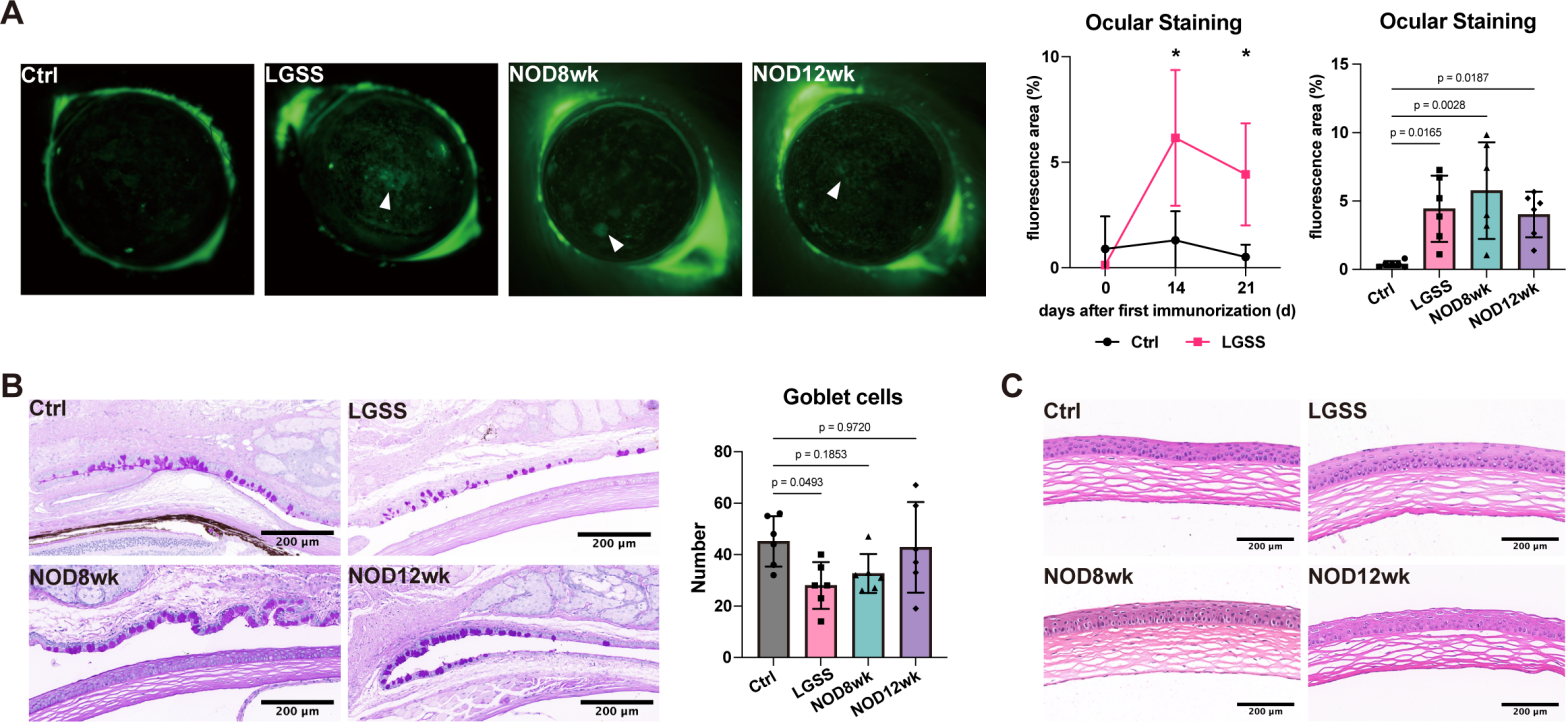
Ocular manifestations in LGSS mice resemble DED. **(A)** Corneal fluorescein staining was performed to evaluate corneal epithelial integrity, and fluorescein scores were analyzed (white triangle indicates epithelial defect areas). **(B)** PAS staining highlighting goblet cells in the conjunctival sac, with quantification of goblet cell density (scale bars: 200 μm). **(C)** HE staining showing corneal morphology and structural changes (n=6 mice/group). (*p<0.05).

Before modeling, mice exhibited minimal corneal epithelial staining, with only a few fluorescein sodium spots visible. After modeling, the LGSS mice showed a significant increase in corneal epithelial staining, with some spots merging into larger areas. Although some recovery was observed by the endpoint of modeling at 21 days, a statistical difference remained. The corneal epithelial damage area was significantly larger in all model groups compared to the control group (**Figure 2A**).

In the control group, PAS staining revealed a uniform distribution of PAS-positive goblet cells across the conjunctival epithelium, indicating normal mucin production and an intact basement membrane. In contrast, the LGSS mice exhibited a reduction in PAS-positive goblet cells, along with disrupted and irregular basement membrane staining, suggesting impaired mucin production and basement membrane integrity. However, the distribution and morphology of goblet cells in 8-week-old and 12-week-old NOD mice showed little difference compared to the control group (**Figure 2B**).

Simultaneously, the corneal structure was examined. The corneal architecture appeared normal across all groups of mice, with no evidence of inflammatory cell infiltration (**Figure 2C**).

### LGSS mice have profound lacrimal gland injuries

**Figure 3:**
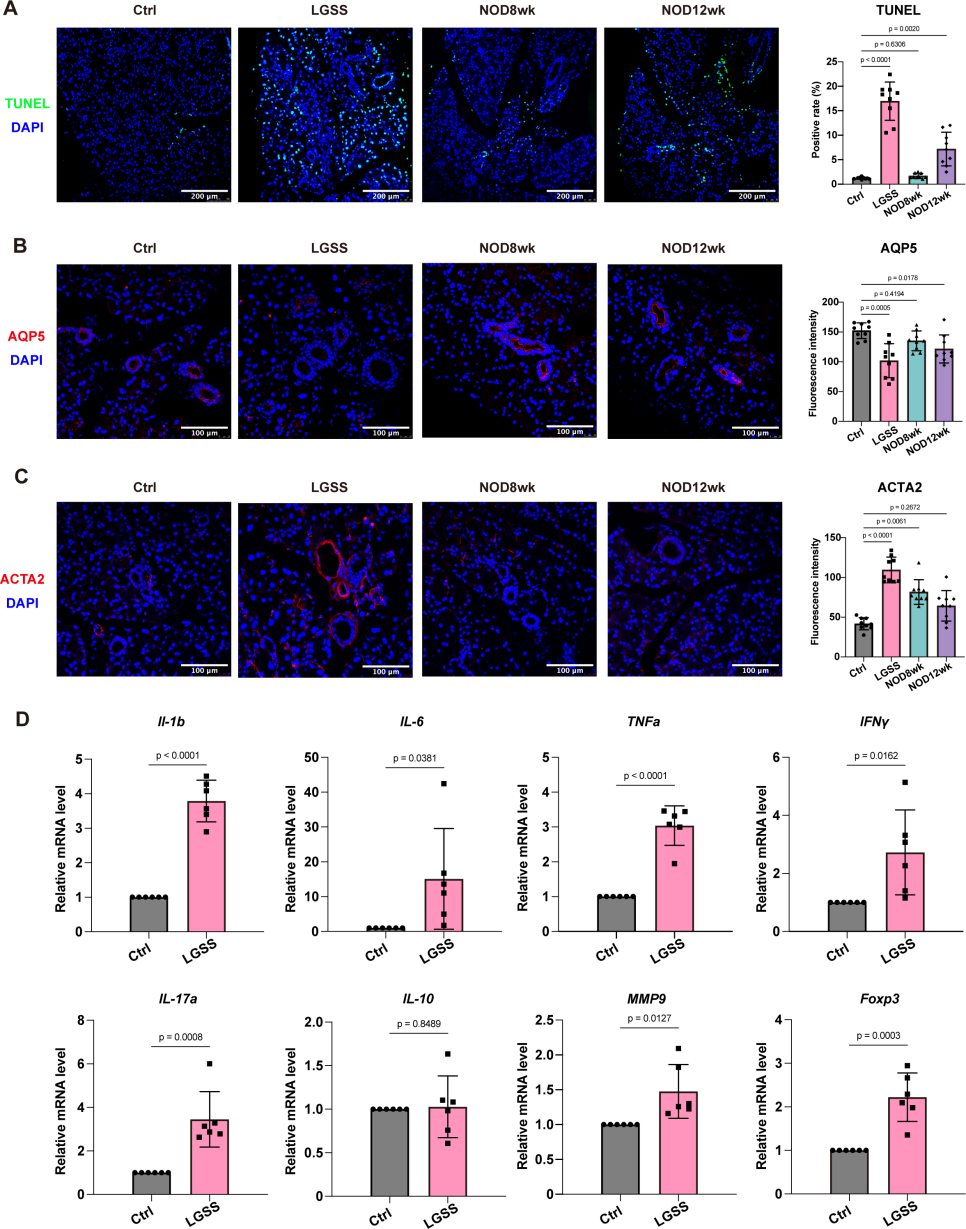
Severe lacrimal gland damage in LGSS mice. **(A)** TUNEL staining of the lacrimal gland indicating the proportion of apoptotic cells (scale bars: 200 μm). **(B, C)** Immunofluorescence staining for AQP5 (glandular duct marker) and ACTA2 (myoepithelial marker), with fluorescence intensity quantified using ImageJ software (scale bars: 100 μm). **(D)** Relative mRNA expression levels of inflammatory cytokines in the lacrimal glands of LGSS mice (n=6 mice/group).

TUNEL staining results of the mouse lacrimal gland tissues indicated a significant increase in apoptotic cell numbers in the lacrimal glands of LGSS and 12-week-old NOD, showing a statistically significant difference compared to the control group, while the number of apoptotic cells in the lacrimal glands of 8-week-old NOD mice was comparable to that in the control group (**Figure 3A**).

Additionally, the expression of AQP5 in the epithelial cells of the lacrimal gland ducts in LGSS mice was significantly lower compared to both control and NOD mice (**Figure 3B**). In contrast, the ACTA2 expression in the myoepithelial cells of LGSS lacrimal glands was significantly higher than in control and NOD mice (**Figure 3C**).

Meanwhile, the mRNA expression levels of selected inflammatory factors in the lacrimal gland tissues of both control and LGSS mice were measured. Results indicated that mRNA levels of *IL-1b*, *IL-6*, *TNF-α*, *IFN-γ*, *IL-17a*, *IL-10*, *MMP9*, and *Foxp3* were significantly elevated in LGSS mice compared to the control group (**Figure 3D**).

### Th1 and Th17 cells are increased in LGSS mice

**Figure 4:**
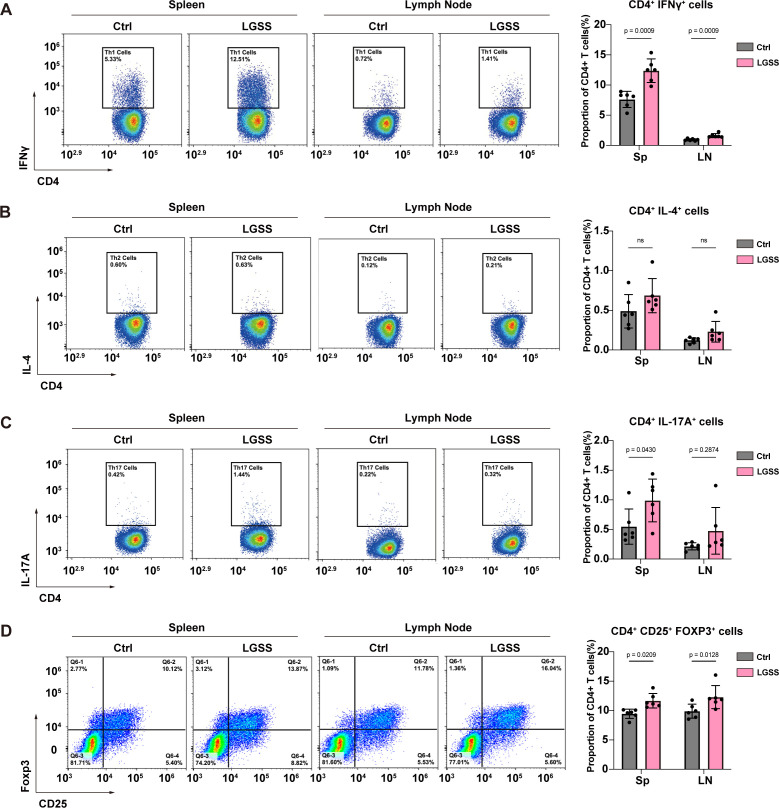
Altered T cell subset distribution in LGSS mice. Flow cytometry analysis of CD4^+^ T cell subsets **(A–D)** of CD4^+^ T cell subsets in spleens and cervical lymph nodes of LGSS and control mice. Data represent the proportion of each subset within CD4^+^ T cells (n=6 mice/group).

Flow cytometry was used to measure the proportions of Th1, Th2, Th17, and Treg cells in the spleen and cervical lymph nodes of control and LGSS mice. Statistical analysis revealed that the proportion of Th1 cells was significantly higher in both the spleen and cervical lymph nodes of LGSS mice compared to the control group. There was no difference in the proportion of Th2 cells between the two groups. The proportion of Th17 cells was significantly different only in the spleen (**Figures 4A–C**). Interestingly, the proportion of Treg cells in both spleen and lymph node of LGSS mice was significantly higher than in the control group (**Figure 4D**).

### Shared and unique molecular signature in LGSS and NOD mice

**Figure 5:**
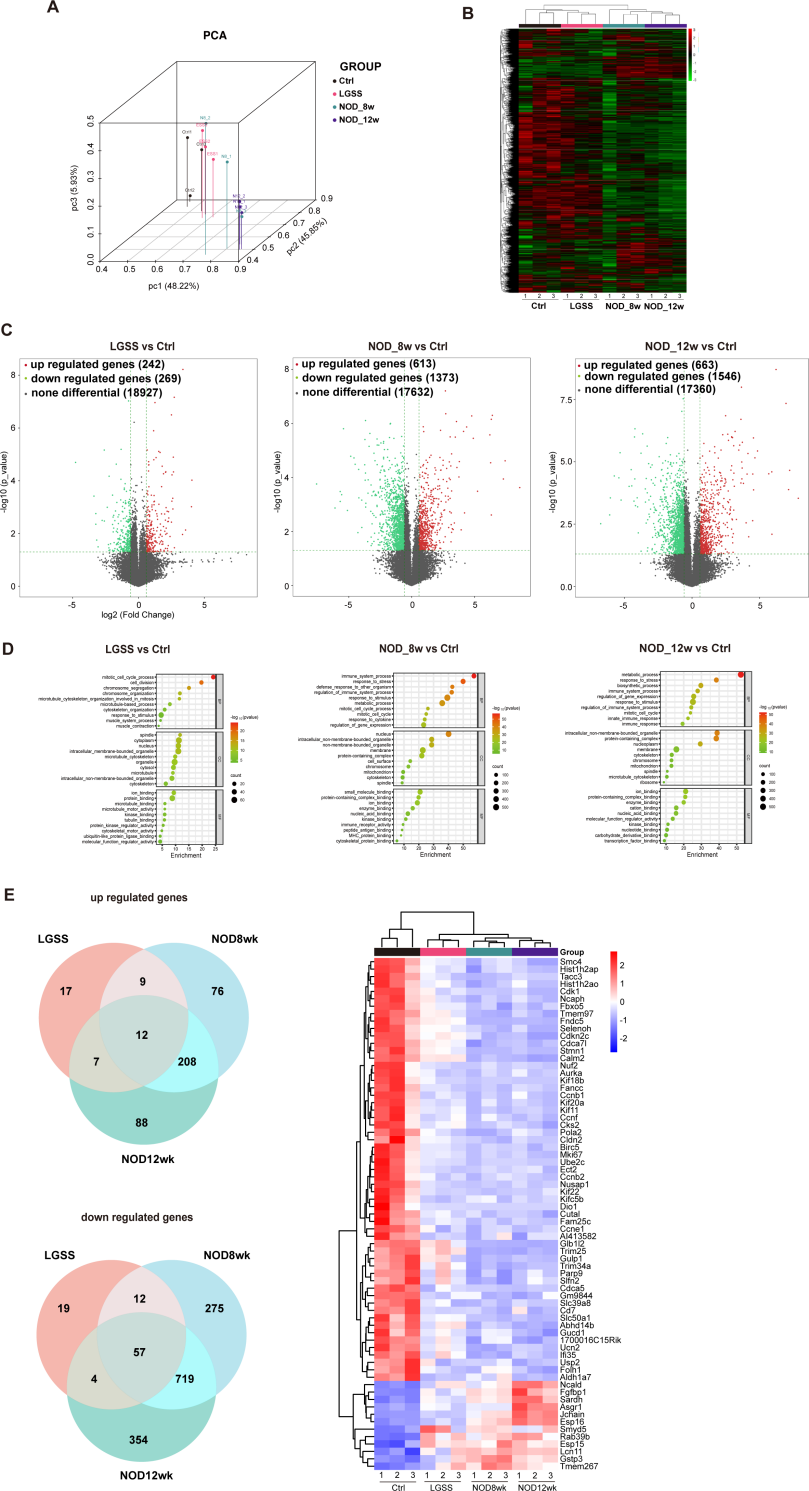
Molecular profiling and comparative analysis of LGSS and NOD mice. **(A)** Principal component analysis of gene expression levels across groups. **(B)** Heatmap showing clustered gene expression profiles among the four groups. **(C)** Volcano plot highlighting differentially expressed genes between LGSS and control mice. **(D)** GO enrichment analysis of differentially expressed genes between LGSS mice and the control group. **(E)** Venn diagram and heatmap illustrating the overlap and distinction of differentially expressed genes between LGSS mice and NOD mice.

To further elucidate the characteristics of the lacrimal glands in LGSS mice, RNA sequencing was performed on the lacrimal glands of the four groups of mice. The three-dimensional principal component analysis (PCA) plot illustrated the variance in gene expression profiles among the four groups: Ctrl, LGSS, 8-week-old, and 12-week-old NOD mice. The axes represent the first three principal components, which collectively account for the majority of the variance in the dataset. The 12-week-old NOD mice exhibit distinct separation from the control group along the three principal components, suggesting significant differences in their gene expression profiles. However, the LGSS mice exhibited a closer proximity to the control group than to the NOD group (**Figure 5A**). The heatmap showed the gene co-expression patterns across the four groups. A distinct separation between the 12-week-old NOD mice compared to the control group in gene expression profiles can be observed (**Figure 5B**).

Subsequently, we utilized volcano plots to illustrate the DEGs in the lacrimal glands of mice from various model groups, providing a clear visualization of the distribution and number of upregulated and downregulated genes (**Figure 5C**). And we conducted GO enrichment analysis and KEGG pathway enrichment analysis on the DEGs between model group and control group (**Figure 5D**). We identified the DEGs that were commonly upregulated and downregulated across the three model groups and visualized their expression patterns using heatmaps (**Figure 5E**).

### Metabolic and cellular process components are involved in novel model of SjD

**Figure 6:**
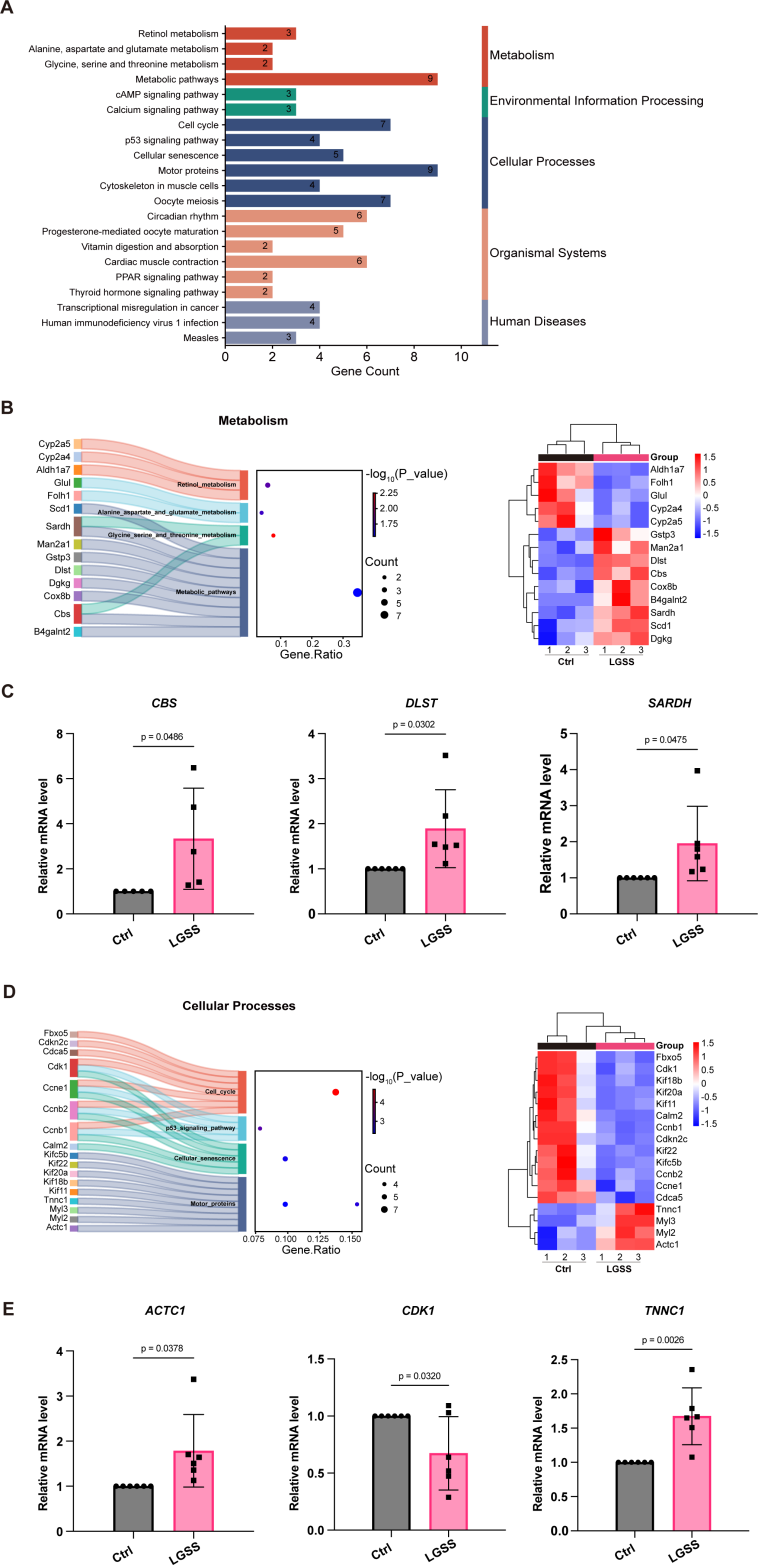
Involvement of metabolic and cellular process pathways in the LGSS model. **(A)** KEGG pathway enrichment classification analysis on the differentially expressed genes between LGSS mice and control mice. **(B)** Sankey dot pathway enrichment and heatmap of differentially expressed genes related to metabolism. **(C)** Relative mRNA expression levels of key genes related to metabolism. **(D)** Sankey dot pathway enrichment and heatmap of differentially expressed genes related to cellular processes. **(E)** Relative mRNA expression levels of key genes related to cellular processes.

We performed KEGG pathway enrichment analysis on the DEGs between LGSS and control group and categorized the pathways based on the annotations from the NCBI database (**Figure 6A**). We identified and listed the DEGs related to metabolism and cellular processes. To confirm the reliability of the sequencing results, we selected several key genes for validation through quantitative qPCR, including genes related with metabolism (*Cbs*, *Dlst*, *Sardh*) and genes associated with cellular processes (*Actc1*, *Tnnc1*). The qPCR results demonstrated high consistency with the sequencing data, supporting the robustness of our findings (**Figures 6B–E**).

## Discussion

The results of this study demonstrate that the LGSS mouse model, induced by lacrimal gland antigens, successfully replicates the key pathological features of SjD, including inflammatory cell infiltration in the exocrine glands, reduced lacrimal and salivary secretion, elevated autoantibodies, and systemic immune activation. Compared to spontaneous models like NOD mice, the LGSS model presents more severe and rapid lacrimal gland damage, making it particularly valuable for studying SjD-DE.

The NOD mice, commonly used to model insulin-dependent diabetes, exhibits significant inflammatory cell infiltration in exocrine glands, and frequently employed in SjD research. To mitigate the impact of diabetes on SjD studies, subtypes like C57BL/6.NOD-*Aec1Aec2* mice have been developed (13, 14). This model shows ocular manifestations including corneal epithelial damage, inflammatory infiltration, and reduced goblet cell numbers (15). Interestingly, at 20 weeks of age, despite inflammation cell infiltration in the lacrimal glands of this model, tear secretion did not decrease but rather increased, suggesting that the ocular symptoms might not be directly related to lacrimal gland pathology (15). This observation aligns with our finding that 8-12-week-old NOD mice exhibit marked corneal epithelial damage prior to significant tear reduction or goblet cell loss. This early damage may be driven by qualitative changes in the tear film (e.g., increased inflammatory cytokines) or early neurotrophic impairment, rather than by aqueous tear deficiency, highlighting the complexity of initial ocular surface insult in SjD-DE. This dissociation of clinical signs is supported by studies in other SjD models, where corneal pathology can manifest independently of tear volume loss (16).

In our study, the inclusion of 8-week-old NOD mice served as a pre-symptomatic baseline, demonstrating that LGSS mice develop lacrimal gland pathology earlier than spontaneous disease onset in NOD mice. Meanwhile, 12-week-old NOD mice were included to represent early disease stages, allowing comparison of disease kinetics (17). While NOD mice eventually develop more severe systemic manifestations (e.g., sialadenitis), their late-onset pathology (typically >20 weeks) complicates mechanistic studies of early SjD-DE. Our model’s rapid induction and lacrimal gland-specific targeting provide a controlled system to study early SjD-DE mechanisms.

The LGSS model showed mild salivary gland lymphocytic infiltration but no significant functional impairment, likely due to its lacrimal gland-specific antigen focus. This contrasts with NOD mice, where systemic autoimmunity drives both lacrimal and salivary gland dysfunction. While this limits the model’s utility for studying full-blown SjD, it offers a precise tool for investigating SjD-DE specific mechanisms, particularly given that many SjD patients present with dry eye before salivary symptoms (18).

In this study, RNA sequencing results from lacrimal gland elucidate key features of the LGSS model. Through analysis of genes commonly expressed in LGSS and NOD mice, we identified several genes critical to the pathogenesis of SjD or other autoimmune diseases, supporting the disease relevance of the LGSS model. For example, *Parp9* is involved in regulating innate immunity and cell death (19), and has also been implicated in the epigenetic regulation of primary SjD (20). *Cdkn2c*, a gene regulating cell cycle progression, modulates B-1a cell numbers and is associated with autoantibody production and the polarization of CD4^+^ T cells toward inflammatory effector functions (21, 22). Additionally, *Ifl35* encodes an IFN-induced protein that plays a critical role in cell proliferation, differentiation, apoptosis, and immune regulation (23). Notably, GO enrichment analysis revealed that the dominant transcriptomic signature in LGSS glands involves epithelial-to-mesenchymal transition and tissue remodeling, rather than broad inflammatory pathways. Importantly, while a low-grade inflammatory response is detectable by sensitive qPCR, the transcriptomic data unequivocally identify tissue remodeling as the dominant and most striking pathological driver in this model. This suggests that the LGSS model drives a rapid, maladaptive repair response, which may underlie the severe and early gland dysfunction observed. This potent induction of tissue-remodeling pathways represents the primary advantage and a key distinction of the LGSS model from classical SjD models, positioning it as a unique and timely tool for investigating fibrosis and aberrant repair in SjD-DE. This unique feature highlights the utility of the LGSS model for studying the fibrotic progression in SjD-DE.

While the LGSS model provides a valuable platform for studying lacrimal gland-specific autoimmunity, certain limitations should be acknowledged. The use of a PBS control is a limitation that precludes distinguishing between the effects of the lacrimal gland antigen and those of the Freund’s incomplete adjuvant. Consequently, our study demonstrates the pathogenic potential of the combined stimulus (antigen + adjuvant), rather than the antigen alone. Future studies incorporating an adjuvant-only control group are essential to deconvolute these contributions and to refine this into a precise antigen-driven model. While this study utilized young NOD mice to validate the induction of disease-like pathology, a dedicated future study is warranted to perform a longitudinal comparison, including aged NOD mice, to precisely map the progression dynamics of lacrimal gland injury in the LGSS model against spontaneous disease. Although we observed systemic immune activation in spleen and lymph nodes, the model’s predominantly lacrimal gland-focused phenotype warrants expanded histopathological evaluation of other target organs (e.g., lungs, kidneys) to assess potential multi-organ involvement. Notably, the absence of significant salivary gland dysfunction, while consistent with the model’s antigen-specific design, highlights an opportunity to explore combined lacrimal-salivary antigen immunization strategies in subsequent studies. This model offers a valuable tool for investigating the pathogenesis of SjD-DE and for developing therapeutic approaches.

## Data Availability

The datasets presented in this study can be found in online repositories. The names of the repository/repositories and accession number(s) can be found below: https://www.ncbi.nlm.nih.gov/, GSE285438.

## References

[1] Foulks GN Forstot SL Donshik PC Forstot JZ Goldstein MH Lemp MA . Clinical guidelines for management of dry eye associated with Sjogren disease. Ocul Surf. (2015) 13:118–32. doi: 10.1016/j.jtos.2014.12.001, PMID: 25881996

[2] Helmick CG Felson DT Lawrence RC Gabriel S Hirsch R Kwoh CK . Estimates of the prevalence of arthritis and other rheumatic conditions in the United States. Part I. Arthritis Rheum. (2008) 58:15–25. doi: 10.1002/art.23177, PMID: 18163481

[3] Pillemer SR Matteson EL Jacobsson LT Martens PB Melton LJ 3rd O’Fallon WM . Incidence of physician-diagnosed primary Sjogren syndrome in residents of Olmsted County, Minnesota. Mayo Clin Proc. (2001) 76:593–9. doi: 10.1016/S0025-6196(11)62408-7 11393497

[4] Whitcher JP Shiboski CH Shiboski SC Heidenreich AM Kitagawa K Zhang S . A simplified quantitative method for assessing keratoconjunctivitis sicca from the Sjgren’s Syndrome International Registry. Am J Ophthalmol. (2010) 149:405–15. doi: 10.1016/j.ajo.2009.09.013, PMID: 20035924 PMC3459675

[5] Liew MSH Zhang M Kim E Akpek EK . Prevalence and predictors of Sjögren’s syndrome in a prospective cohort of patients with aqueous-deficient dry eye. Br J Ophthalmol. (2016) 96:1498. doi: 10.1136/bjophthalmol-2012-301767, PMID: 23001257

[6] Hu Y Nakagawa Y Purushotham KR Humphreysbeher MG . Functional changes in salivary glands of autoimmune disease-prone NOD mice. Am J Physiol. (1992) 263:607–14. doi: 10.1152/ajpendo.1992.263.4.E607, PMID: 1415679

[7] Kiripolsky J Shen L Liang Y Li A Suresh L Lian Y . Systemic manifestations of primary Sjögren’s syndrome in the NOD.B10Sn-H2(b)/J mouse model. Clin Immunol. (2017) 183:225–32. doi: 10.1016/j.clim.2017.04.009, PMID: 28526333 PMC6082154

[8] Lin X Rui K Deng J Tian J Wang X Wang S . Th17 cells play a critical role in the development of experimental Sjögren’s syndrome. Ann Rheum Dis. (2015) 74:1302–10. doi: 10.1136/annrheumdis-2013-204584, PMID: 24573745

[9] Nguyen C Singson E Kim JY Cornelius JG Attia R Doyle ME . Sjögren’s syndrome-like disease of C57BL/6.NOD-Aec1 Aec2 mice: gender differences in keratoconjunctivitis sicca defined by a cross-over in the chromosome 3 Aec1 locus. Scand J Immunol. (2006) 64:295–307. doi: 10.1111/j.1365-3083.2006.01828.x, PMID: 16918699

[10] Ophthalmology. AfRiVa . Statement for the Use of Animals in Ophthalmic and Vision Research (2023). Available online at: https://www.arvo.org/about/policies/arvo-statement-for-the-use-of-animals-in-ophthalmic-and-vision-research/ (Accessed June 30, 2024).

[11] Zhang P Tian L Bao J Li S Li A Wen Y . Isotretinoin impairs the secretory function of meibomian gland via the PPARγ Signaling pathway. Invest Ophthalmol Visual Sci. (2022) 63:29–. doi: 10.1167/iovs.63.3.29, PMID: 35353124 PMC8976919

[12] Li Y Tian L Li S Chen X Lei F Bao J . Disrupted mitochondrial transcription factor A expression promotes mitochondrial dysfunction and enhances ocular surface inflammation by activating the absent in melanoma 2 inflammasome. Free Radical Biol Med. (2024) 222:106–21. doi: 10.1016/j.freeradbiomed.2024.05.032, PMID: 38797339

[13] Cha S Nagashima H Brown VB Peck AB Humphreys-Beher MG . Two NOD Idd-associated intervals contribute synergistically to the development of autoimmune exocrinopathy (Sjögren’s syndrome) on a healthy murine background. Arthritis Rheumatol. (2014) 46:1390–8. doi: 10.1002/art.10258, PMID: 12115247

[14] Peck AB Nguyen CQ Ambrus JL . Upregulated chemokine and rho-GTPase genes define immune cell emigration into salivary glands of sjögren ‘s syndrome-susceptible C57BL/6.NOD-aec1Aec2 mice. Int J Mol Sci. (2021) 13. doi: 10.3390/ijms22137176, PMID: 34281229 PMC8267620

[15] In-Cheon Y Fang B Volpe EA De PCS Pflugfelder SC . Age-related conjunctival disease in the C57BL/6.NOD-aec1Aec2 mouse model of sjgren syndrome develops independent of lacrimal dysfunction. Invest Ophthalmol Vis. (2015) 56:2224–33. doi: 10.1167/iovs.14-15668, PMID: 25758816 PMC4687887

[16] Turpie B Yoshimura T Gulati A Rios JD Dartt DA Masli S . Sjgren’s syndrome-like ocular surface disease in thrombospondin-1 deficient mice. Am J Pathol. (2009) 175:1136–47. doi: 10.2353/ajpath.2009.081058, PMID: 19700744 PMC2731132

[17] Winer S Astsaturov I Cheung R Tsui H Dosch HM . Primary Sjogren’s syndrome and deficiency of ICA69. Lancet. (2002) 360:1063–9. doi: 10.1016/S0140-6736(02)11144-5, PMID: 12383988

[18] Stapleton F Alves M Bunya VY Jalbert I Lekhanont K Malet F . TFOS DEWS II epidemiology report. Ocul Surf. (2017) 15:334–65. doi: 10.1016/j.jtos.2017.05.003, PMID: 28736337

[19] Penelope K Martina BEE Henk-Jan VDH Fatiha ZB Wilfred VI Jouke R . Analysis of mouse brain transcriptome after experimental Duvenhage virus infection shows activation of innate immune response and pyroptotic cell death pathway. Front Microbiol. (2018) 9:397. doi: 10.3389/fmicb.2018.00397, PMID: 29615985 PMC5869263

[20] Imgenberg-Kreuz J Sandling JK Almlof JC Nordlund J Signer L Norheim KB . Genome-wide DNA methylation analysis in multiple tissues in primary Sjogren’s syndrome reveals regulatory effects at interferon-induced genes. Ann Rheum Dis. (2016) 75:2029–36. doi: 10.1136/annrheumdis-2015-208659, PMID: 26857698 PMC5099203

[21] Zhiwei X Laurence M . Contribution of B-1a cells to systemic lupus erythematosus in the NZM2410 mouse model. Ann New York Acad Sci. (2016) 1362:215–23. doi: 10.1111/nyas.12607, PMID: 25728381 PMC4550580

[22] Ju J Xu J Zhu Y Fu X Xu Z . A variant of the histone-binding protein sNASP contributes to mouse lupus. Front Immunol. (2019) 10:637. doi: 10.3389/fimmu.2019.00637, PMID: 31001259 PMC6454087

[23] Chen S Haolong L Zhan H Zeng X Yuan H Li Y . Identification of hub biomarkers and immune cell infiltration in polymyositis and dermatomyositis. Aging. (2022) 14:4530–55. doi: 10.18632/aging.204098, PMID: 35609018 PMC9186768

