# Supplementary material for: A novel murine model of Sjögren’s disease using lacrimal autoantigen
Source: Front Immunol. 2026 Feb 13;17:1586519. doi: 10.3389/fimmu.2026.1586519 (PMC12946062; doi:10.3389/fimmu.2026.1586519)
Supplement: Supplementary file 1 [file DataSheet1.pdf]

Supplementary Table 1. **Primer sequences used in the study for RT-qPCR**

Name	5’-sequence-3’
IL-1b	F: TTC AGG CAG GCA GTA TCA CTC<br>R: GAA GGT CCA CGG GAA AGA CAC
IL-6	F: GTG AAA GCA GCA AAG AGG C<br>R: TTT CAC CAG GCA AGT CTC C
TNF-a	F: CCT CTC TCT AAT CAG CCC TCT G<br>R: GAG GAC CTG GGA GTA GAT GAG
IFNg	F: GCTTTGCAGCTCTTCCTCAT<br>R: CGACTCCTTTTCCGCTTCCT
IL-17a	F: CCT GGC GGC TAC AGT GAA G<br>R: TTT GGA CAC GCT GAG CTT TG
IL-10	F: GCT CTT ACT GAC TGG CAT GAG<br>R: CGC AGC TCT AGG AGC ATG TG
MMP9	F: TCT ATG GTC CTC GCC CTG AA<br>R: CAT CGT CCA CCG GAC TCA AA
Foxp3	F: CCC TGC CCT TGA CCT CAA<br>R: GCC TCA GTC TCA TGG TTT TGG
CBS	F: GTG GCG TCT GCG TGT TCA AG<br>CGG AGG ATG GCG ATG GTG TC
DLST	F: TGA TGT ATG TAG CCC TGA CCT ATG AC<br>R: CTT GGA TCT TCT ACT GCT GCC TTG
SARDH	F: TTC TTC CTT GGC TGT GGC TTC AAC<br>R: TGG TCC GTG AGC GAG TGA TGG
ACTC1	F: CCA AGG CCA ACC GTG AGA AG<br>R: GAG ACA GCA CTG CCT GGA TG
TNNC1	F: CTG AGG AGG AGC TGT CGG AT<br>R: AAT GGT CTC ACC TGT GGC CT
18S	F: TGA GAA ACG GCT ACC ACA TCC<br>R: CAC CAG ACT TGC CCT CCA AT
